# Potential Prognostic Biomarkers of OSBPL Family Genes in Patients with Pancreatic Ductal Adenocarcinoma

**DOI:** 10.3390/biomedicines9111601

**Published:** 2021-11-03

**Authors:** Cheng-Wei Chou, Yu-Hsiu Hsieh, Su-Chi Ku, Wan-Jou Shen, Gangga Anuraga, Hoang Dang Khoa Ta, Kuen-Haur Lee, Yu-Cheng Lee, Cheng-Hsien Lin, Chih-Yang Wang, Wei-Jan Wang

**Affiliations:** 1 Division of Hematology/Medical Oncology, Department of Internal Medicine, Taichung Veterans General Hospital, Taichung 40705, Taiwan; 1983ccwei@gmail.com (C.-W.C.); briefsummer@gmail.com (C.-H.L.); 2 Department of Biological Science and Technology, China Medical University, Taichung 40676, Taiwan; sandy2710265@gmail.com; 3 Graduate Institute of Cancer Biology and Drug Discovery, College of Medical Science and Technology, Taipei Medical University, Taipei 11031, Taiwan; b101104152@tmu.edu.tw (S.-C.K.); g.anuraga@unipasby.ac.id (G.A.); d621109004@tmu.edu.tw (H.D.K.T.); khlee@tmu.edu.tw (K.-H.L.); 4 Ph.D. Program for Cancer Molecular Biology and Drug Discovery, College of Medical Science and Technology, Taipei Medical University and Academia Sinica, Taipei 11031, Taiwan; 5 School of Medicine, College of Medicine, Taipei Medical University, Taipei 11031, Taiwan; 6 Graduate Institute of Biomedical Sciences, College of Medicine, China Medical University, Taichung 40402, Taiwan; wshen@cmu.edu.tw; 7 Department of Statistics, Faculty of Science and Technology, Universitas PGRI Adi Buana, Surabaya 60234, East Java, Indonesia; 8 Graduate Institute of Medical Sciences, College of Medicine, Taipei Medical University, Taipei 11031, Taiwan; yclee0212@tmu.edu.tw; 9 Research Center for Cancer Biology, China Medical University, Taichung 40676, Taiwan

**Keywords:** pancreatic ductal adenocarcinoma, biomarker, OSBPL2, OSBPL3, OSBPL5, OSBPL6, OSBPL7, OSBPL8, OSBPL9, OSBPL10, OSBPL11

## Abstract

Pancreatic ductal adenocarcinoma (PDAC) is a highly fatal malignancy with poor survival outcomes. In addition, oxysterol-binding protein-like (OSBPL) family members are reported to be involved in lipid binding and transport and play critical roles in tumorigenesis. However, relationships between PDAC and OSBPL family members have not comprehensively been elucidated. In this study, we used the Oncomine and GEPIA 2 databases to analyze OSBPL transcription expressions in PDAC. The Kaplan–Meier plotter and TIMER 2.0 were used to assess the relationships between overall survival (OS) and immune-infiltration with OSBPL family members. Co-expression data from cBioPortal were downloaded to assess the correlated pathways with OSBPL gene family members using DAVID. The expressions of OSBPL3, OSBPL8, OSBPL10, and OSBPL11 were found to be highly upregulated in PDAC. Low expressions of OSBPL3, OSBPL8, and OSBPL10 indicated longer OS. The functions of OSBPL family members were mainly associated with several potential signaling pathways in cancer cells, including ATP binding, integrin binding, receptor binding, and the renin-angiotensin system (RAS) signaling pathway. The transcription levels of OSBPL gene family members were connected with several immune infiltrates. Collectively, OSBPL family members are influential biomarkers for the early diagnosis of PDAC and have prognostic value, with the promise of precise treatment of PDAC in the future.

## 1. Introduction

Pancreatic ductal adenocarcinoma (PDAC) is the most lethal malignancy among all types of cancers—with a 5-year survival rate of 5%—because it is not easily diagnosed at an early stage. In an earlier published study, it was estimated that PDAC would become the second-deadliest cancer in the United States by 2025. Although several medical interventions have made excellent progress, including surgery and chemotherapy, PDAC still has poor treatment outcomes and a high mortality worldwide (http://www.iacr.com.fr/, accessed on 15 April 2021). Therefore, finding novel and robust biomarkers for prognoses in patients with PDAC is crucial.

The oxysterol-binding protein (OSBP)-like (OSBPL) protein families are composed of nine members and which are intracellular lipid-binding/transport proteins that are necessary for lipid transport and maintaining a balance of cholesterol in the body [1]. Their structures include a lipid-binding domain and membrane-targeting determinants (an N-terminal pleckstrin homology (PH) domain) [2], which are typically positioned at the membrane contact sites and are used to exchange inter-organelle small molecules and information [1,3,4]. Moreover, due to their critical roles in cellular functions, including migration, proliferation, and vesicular trafficking [5], they are involved in a variety of human malignancies. For instance, the upregulation of OSBPL3 can promote colorectal cancer tumorigenesis [2], and OSBPL5 may become a potential indicator of diagnosing of prostate cancer [6,7]. OSBPL2, OSBPL7, and OSBPL8 were also reported to be overexpressed in cholangiocarcinoma [8]. OSBPL10 may become a prognostic marker for gastric cancer [9] and B-cell lymphoma [10]. OSBPL11 is a potential marker for hepatocellular carcinoma [11]. An overall analysis of the functions of OSBPL families for prognoses in patients with PDAC still needs to be explored.

In this study, we systemically analyzed the mRNA transcription levels of OSBPL mambers and their prognostic value in terms of survival patients with PDAC. Using multiple bioinformatic analyses, we revealed that the molecular biological functions of OSBPLs family genes in PDAC, especially OSBPL3, might provide a novel mechanism in the tumorigenesis and cancer immunology of PDAC. Our findings could be clinically valuable for developing novel therapeutic strategies, prognosis assessments, and biomarkers for immunotherapy of PDAC.

## 2. Materials and Methods

### 2.1. Oncomine Gene Analysis of OSBPL Family Members in PDAC

The Oncomine bioinformatics tool contains cancer microarray data and a platform for integrating network data analyses (http://www.oncomine.org/, accessed on 15 April 2021) that aims to promote whole-genome expression analyses and compare transcription-level data in target cancers with respective normal tissues [12]. The individual gene transcription levels of OSBPL2~OSBPL11 were analyzed using Oncomine platform. In this research, we compared messenger (m)RNA expression levels in cancer tissues with those in normal tissues and set a cut-off of *p* = 0.01 and a 1.5-fold change [13,14,15,16,17].

### 2.2. The Gene Expression Profiling Interactive Analysis 2 (GEPIA 2) Database Analysis of Clinicopathological Factors of the OSBPL Gene Family

Introduced in 2017, GEPIA 2 is an updated and enhanced version of a website server (http://gepia2.cancer-pku.cn/, accessed on 15 April 2021), which analyzes the RNA sequencing expression data of 198,619 isoforms and 84 cancer subtypes from The Cancer Genome Atlas (TCGA) and the Genotype-Tissue Expression projects using a standard processing pipeline. GEPIA 2 was expanded from the gene level to the transcription level for quantifying gene-level expressions. It thus offers a variety of functions, including tumor/normal tissue expression analyses, cancer types, pathological stages, patient survival analyses, and correlation analyses [18,19,20].

### 2.3. Cancer Cell Line Encyclopedia (CCLE) Analysis

Since cancer cell lines are essential tools for further scientific research, we used the CCLE to explore the OSBPL family transcription levels in PDAC cell lines [21,22]. The CCLE is a public access database containing 947 human cancer cell lines, including genomic data, analyses, and visualization. Log2-transformed OSBPL family expression values were exported and plotted using a heatmap format, as we previously described [23,24,25].

### 2.4. Kaplan–Meier (KM) Plotter Evaluates the Influence of Expressions of OSBPL Family Transcription Levels in PDAC

The Kaplan–Meier plotter contains information about the associations of gene expressions with the survival of patients with many kinds of cancers (www.kmplot.com, accessed on 15 April 2021) [26]. We analyzed the prognostic merits of the target genes [27] with the KM plotter using median values, hazard ratios (HRs) >1, and a log rank of <0.05 [28,29,30,31,32,33].

### 2.5. Analysis of Genetic Alterations by cBioPortal

Information regarding OSBPL alterations in PDAC was assessed using cBioPortal (http://www.cbioportal.org, accessed on 15 April 2021) [34]. We selected TCGA database, a publicly available resource that contains multidimensional cancer genomics data. Nowadays, cBioPortal provides data from more than 5000 tumor samples from 20 cancer studies. It offers mutation data, copy number alterations, protein and phosphoprotein levels, microarray-based and RNA-sequencing-based mRNA expression changes, and DNA methylation values [35].

### 2.6. GeneMANIA Analysis for Functions and Interactions of OSBPL Gene Family Members

GeneMANIA (http://gepia.cancer-pku.cn/index.html, accessed on 15 April 2021) is an online tool that offers information on networks, including shared protein domains, physical interactions, and the co-expression and function of target genes [36].

### 2.7. STRING Analysis for OSBPL Family Members and Other Related Proteins

The goals of the STRING database (http://string-db.org/, accessed on 15 April 2021) are to collect and integrate information by predicting protein-protein interaction (PPI) data for multiple organisms. In this study, we investigated OSBPL gene family members and their connected proteins of PPI networks [37].

### 2.8. Co-Expression Materials Obtained from DAVID through cBioPortal

The Database for Annotation, Visualization and Integrated Discovery (DAVID) (http://www.linkedomics.org/, accessed on 15 April 2021) is a useful bioinformatics tool for evaluating the relevance of target genes. In this research, we acquired the co-expression data of OSBPL family members from the cBioPortal platform, and performed analyses using gene ontology (GO), Kyoto Encyclopedia of Genes and Genomes (KEGG), and BIOCARTA of DAVID to identify closely correlated neighbor genes of related pathways [36]. The second part determined the biological processes, disease biomarker networks, and breast neoplasm cell-cell signaling pathways using a MetaCore analysis. Furthermore, a GO analysis was also implemented to describe genes and gene products from three categories: cell compositions, molecular functions (MFs), and biological processes (BPs) [38,39,40].

### 2.9. Analysis of Protein Expressions in Clinical Human Specimens

The OSBPL family protein expressions were further evaluated using the publicly available Human Protein Atlas (HPA) platform, which contains images of tissue microarrays labeled with antibodies alongside 11,250 human proteins. These microarrays contain sections from 46 normal human tissues and more than 20 types of human cancer [41,42,43].

### 2.10. Tumor Immune Estimation Resource (TIMER) 2.0 contains Materials of Immune-Infiltration of OSBPL Gene Family Members

We used TIMER 2.0 (http://timer.comp-genomics.org/, accessed on 15 April 2021) to explore the infiltration levels of immune cells in 31 tumor types in more than 10,000 samples extracted from the TCGA database [44,45]. The TIMER 2.0 database was used to determine abundances of tumor infiltrates based on gene expression analyses. The DiffExp module with default parameters was used to obtain the different expression levels of OSBPL family genes in normal and tumor tissues. B cells, cluster of differentiation 8-positive (CD8^+^) T cells, CD4^+^ T cells, neutrophils, macrophages, and dendritic cells (DCs) were selected as the test types.

### 2.11. Statistical Analysis

We utilized TCGA Pan-Cancer Atlas, a dataset from cBioPortal (http://www.cbioportal.org, accessed on 15 April 2021), to obtain patients’ data and query gene expression of different OSBPL family members. For the survival analysis, the KM plotter was applied; with all default settings, the overall survival (OS) was preferred, with the J best probe set and median cut-off values. The log-rank *p* < 0.05 was considered to be statistically significant.

## 3. Results

### 3.1. OSBPL Gene Expressions in PDAC

**Figure 1:**
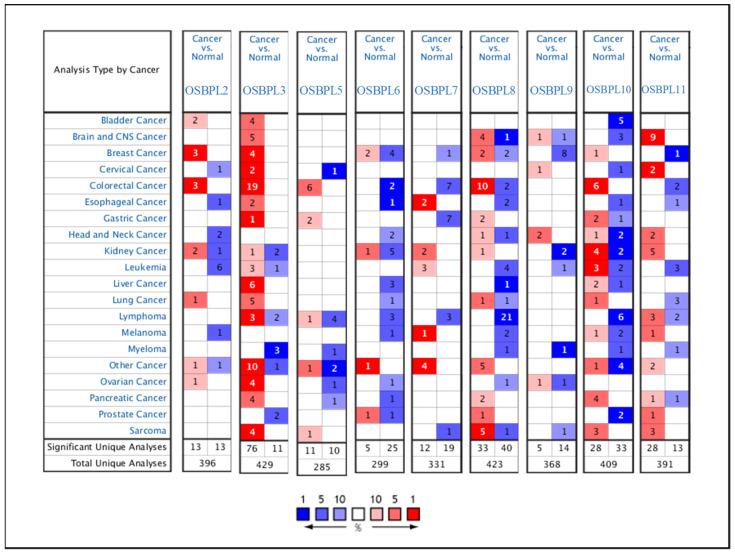
Gene transcript expressions of proteins secreted by oxysterol-binding protein (OSBP)-like (OSBPL) family members in different types of cancers from the Oncomine database. Statistically significant differences in the mRNA overexpression of OSBP family genes are shown in red, and differences in downregulation are shown in blue.

OSBPL family members are widely expressed in different tumor tissues, but there are no reports linking OSBPL family genes with PDAC. Therefore, we first identified the transcriptional levels of OSBPL family members the levels in normal tissues and cancer tissues by an Oncomine bioinformatic analysis (Figure 1). The data demonstrated that OSBPL3, OSBPL8, OSBPL10, and OSBPL11 were overexpressed in pancreatic cancer tissues. In the PDAC dataset of Segara et.al [46], the transcriptional levels of OSBPL3 and OSBPL8 in tumors were higher than in normal samples with a multiple of change of 2.872. In a dataset of Pei et.al [47], the expression of OSBPL3 was 3.571-fold higher in PDAC than in normal tissues, and were respectively 3.221- and 1.952-fold higher in the Badea et.al. [48] and Iacobuzio-Donahue et.al datasets [49]. The OSBPL8 expression level was also higher in PDAC with a multiple of change of 2.119 in a the dataset of Badea [50]. As for OSBPL10, there were 3.438- and 3.527-fold overexpression levels in pancreatic carcinoma and 4.158- and 4.814- fold overexpression levels in PDAC, respectively. for OSBPL11, there was an increase in pancreatic carcinoma with a multiple of change of 1.509. The Oncomine analysis indicated that there were no significant differences between the transcriptional levels of OSBPL2, OSBPL7, and OSBPL9. There were lower transcriptional levels than in normal samples with multiples of change of −2.964 for OSBPL5 [50] and −4.006 for OSBPL6 [51] (Supplementary Table S1).

### 3.2. Associations of Transcription Levels with Clinicopathological Factors of OSBPL Family Members in Patients with PDAC

**Figure 2:**
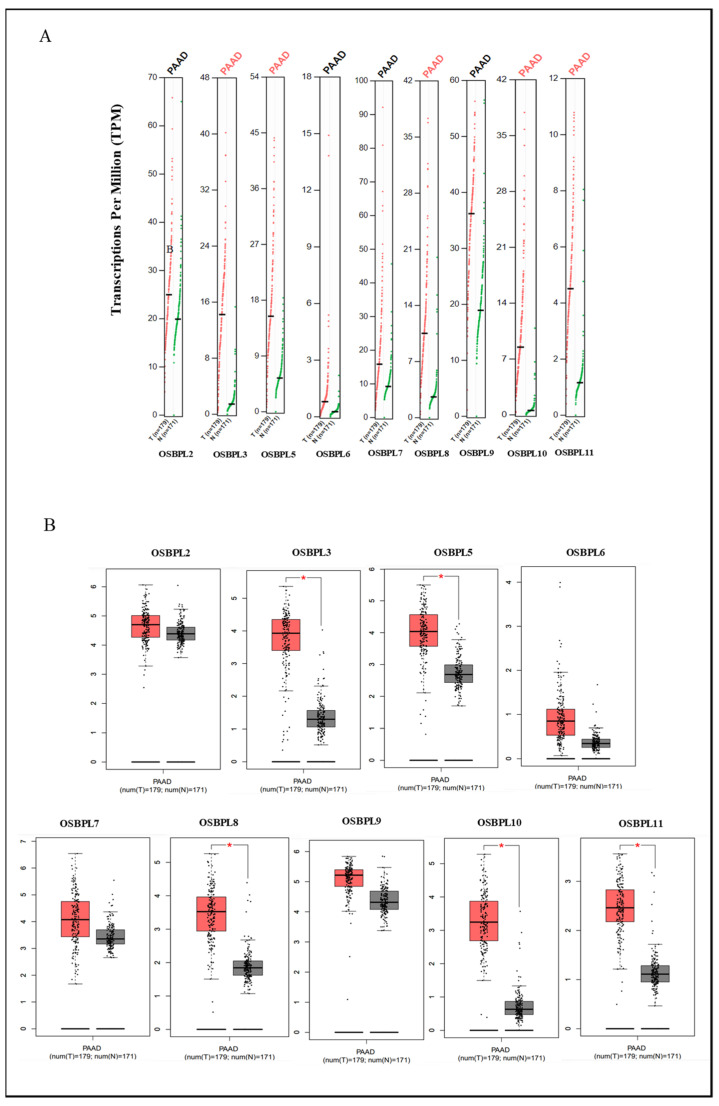
Transcription levels of oxysterol-binding protein (OSBP)-like (OSBPL) family members in pancreatic ductal adenocarcinoma (PDAC) patients. (**A**) Expressions of OSBPL members in PDAC) patients and normal tissue via the GEPIA 2 platform. The q-value cut-off was set to 0.01. (**B**) The red star in the pictures indicates a significant difference between PDAC and normal tissues; the *p*-value cut-off was set to 0.01 via the GEPIA 2 platform. PAAD in (**B**) is equal to PDAC. * *p* < 0.05.

**Figure 3:**
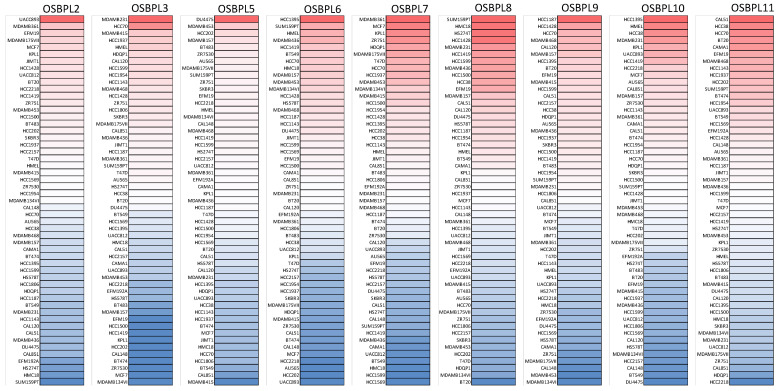
Heatmap plots representing oxysterol-binding protein (OSBP)-like (OSBPL) family gene expression levels in all pancreatic ductal adenocarcinoma (PDAC) cell lines acquired from the CCLE database. The upturned blocks in red indicate overexpression, while the lower blocks in blue indicate underexpression.

Furthermore, we used the GEPIA dataset to compare the transcriptional mRNA levels of OSBPL family members between PDAC and normal pancreatic samples. The transcription levels of OSBPL3, OSBPL5, OSBPL8, OSBPL10, and OSBPL11 in PDAC were found to significantly differ from the levels in normal samples, which indicated that patients with PDAC had higher expressions of OSBPL3, OSBPL5, OSBPL8, OSBPL10, and OSBPL11 according to a clinicopathological analysis; however, the expressions of OSBPL2, OSBPL6, OSBPL7, and OSBPL9 in PDAC were not higher than those in normal samples (Figure 2A), and a box plot also indicated similar results (Figure 2B). In addition, we also explored expressions of OSBPL family genes in a variety of PDAC cell lines in the CCLE (Figure 3).

### 3.3. Prognostic Values of OSBPL Family Members in PDAC

**Figure 4:**
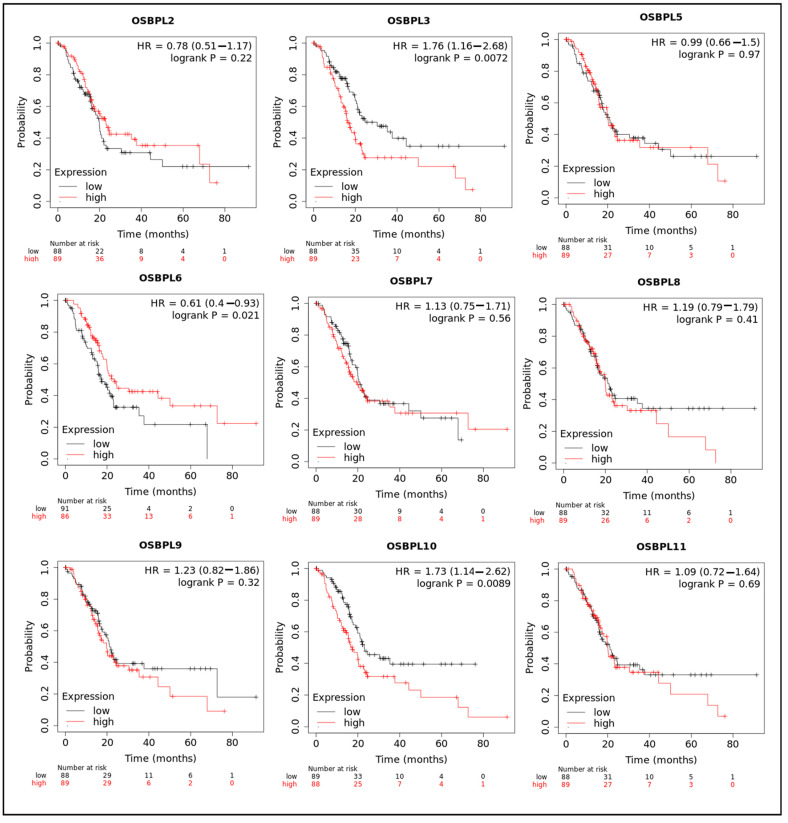
Prognostic values of transcription levels of oxysterol-binding protein (OSBP)-like (OSBPL) family members in pancreatic ductal adenocarcinoma (PDAC) patients in terms of overall survival (OS). Patients were divided based on median values via the Kaplan–Meier plotter platform.

**Figure 5:**
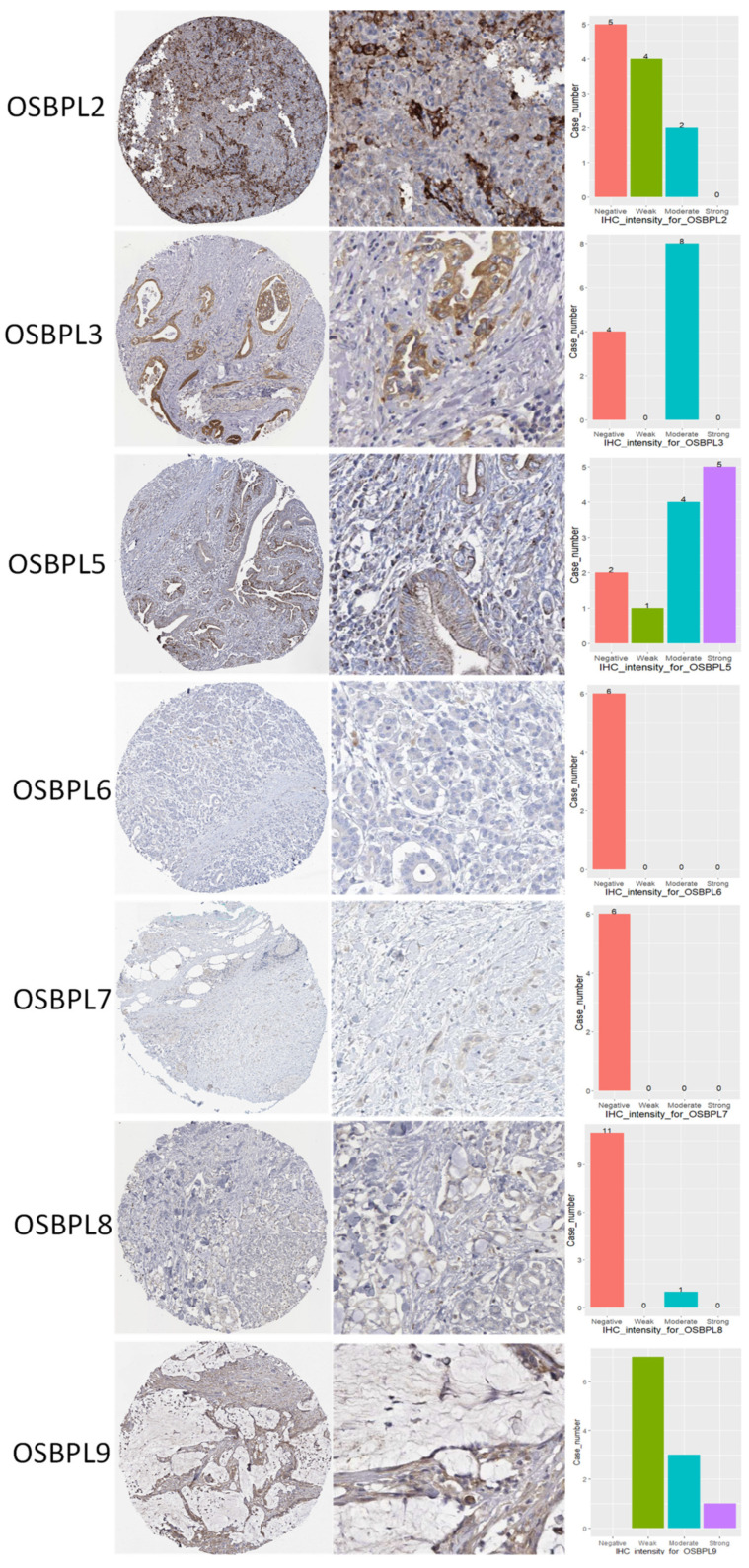
Protein expression levels of oxysterol-binding protein (OSBP)-like (OSBPL) family members in clinical pancreatic ductal adenocarcinoma (PDAC) tissues. Protein expression data of OSBPL family members in cancer specimens were acquired from the Human Protein Atlas. Bar charts show the IHC staining intensities of OSBPL family proteins from the PDAC dictionary.

**Figure 6:**
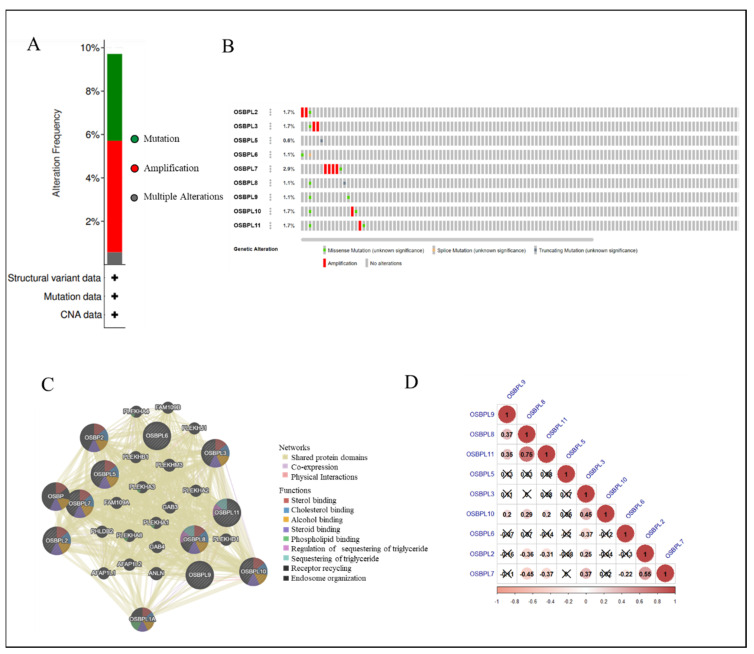
Genetic alterations and an association analysis of oxysterol-binding protein-like (OSBPL) in pancreatic ductal adenocarcinoma (PDAC) via cBioPortal and GeneMANIA Platform. (**A**) Summary of genetic alterations in OSBPL members with PDAC. (**B**) Individuals showed genetic alterations in OSBPL family genes with PDAC. (**C**) Gene–gene interaction networks among various OSBPL family members in PDAC. (**D**) Correlations between different OSBPL family members in TCGA PDAC patients; insignificant correlations are indicated by crosses.

The KM plotter was used to evaluate the effects of OSBPL family genes on PDAC survival statuses (Figure 4). Patients with low transcription levels of OSBPL3 (*p* = 0.0072; hazard ratio (HR) = 1.76) and OSBPL10 (*p* = 0.0089; HR = 1.73) were significantly correlated with longer overall survival (OS). On the contrary, a high expression of OSBPL6 (*p* = 0.021; HR = 0.61) was correlated with a longer OS. OSBPL2, OSBPL5, OSBPL7, OSBPL8, OSBPL9, and OSBPL11 demonstrated no significance in OS (Supplementary Table S2). We next further explored potential roles of OSBPL family genes in clinical PDAC specimens; the data demonstrated that OSBPL2, OSBPL3, OSBPL5, and OSBPL9 had moderate expression levels and OSBPL5 and OSBPL9 had further strong positive expression levels in PDAC specimens (Figure 5). These results suggested that certain OSBPL family members could be biomarkers for the progression of PDAC in patients. The results indicated that the *PLEKH* gene family, *GAB* gene family, *ANLN*, etc., were associated with *OSBPL* gene family members (Figure 6C). Furthermore, we calculated the correlation coefficients between OSBPL members based on their mRNA expression levels using Pearson’s correlation analysis. Based on the correlation plot, the results showed relatively large and significant positive correlations of the OSBPL2 gene with OSBPL3 and OSBPL7; OSBPL3 with OSBPL2, OSBPL7, and OSBPL10; and OSBPL8 with OSBPL9, OSBPL10, and OSBPL11 (Figure 6D).

### 3.4. Analyses of Genetic Alterations, Co-Expressions, and Interactions of OSBPL Family Members in PDAC

To further explore, visualize, and analyze the genomic data, we applied OSBPL family proteomic data to the cBioPortal online bioinformatics tool for PanCancer (TCGA). Based on the cohort (Figure 6A) demonstrated that among 175 PDAC patients, OSBPL members were altered in 17 samples and those alterations included mutations and amplifications. According to the outcome, the rates of genetic alternations of OSBPL members in patients with PDAC ranged from the highest rate of 2.9% for OSBPL7 to the lowest rate of 0.6% for OSBPL5. The remaining rates of the OSBPL family genes were 1.7% for OSBPL2, OSBPL3, OSBPL10, and OSBPL11 and 1.1% for OSBPL6, OSBPL8, and OSBPL9 (Figure 6B). In addition, we also used GeneMANIA to explore the associations of OSBPL members with other genes and networks, including the shared protein domains, co-expressions, and physical interactions, and functions (e.g., sterol binding, alcohol binding, phospholipid, etc.) of OSBPL family members.

### 3.5. PPIs and Co-Expression for Pathway Enrichment Analysis of OSBP Family Members in Patients with PDAC

**Figure 7:**
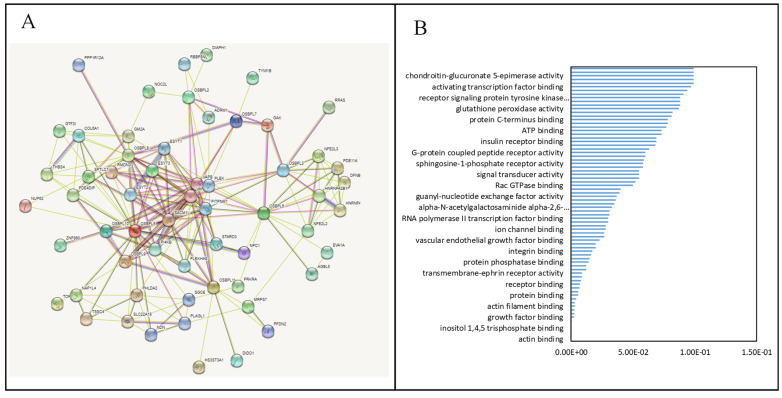
Protein–protein interaction (PPI) networks and pathways for the co-expression of oxysterol-binding protein (OSBP)-like (OSBPL) family members in pancreatic ductal adenocarcinoma (PDAC). (**A**) PPI networks of various proteins and OSBPL family members. Colors of the lines express the types of interactions by STRING. (**B**) Bar plot of gene ontology (GO) abundances in molecular function (MF)-enriched terms by cBioPortal and DAVID.

**Figure 8:**
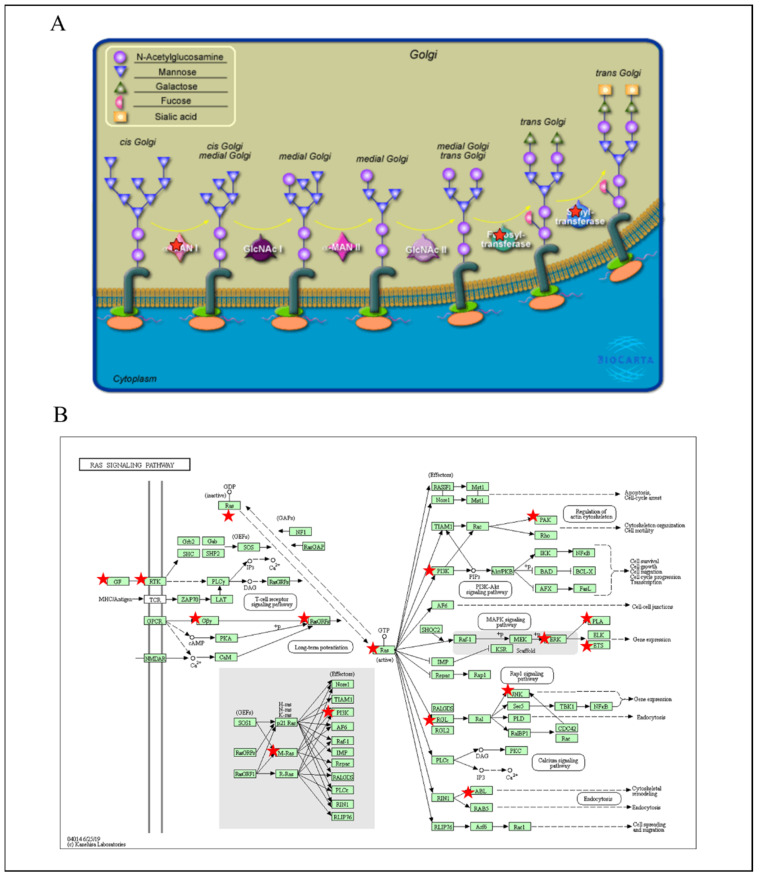
Pathway analysis for the co-expression of oxysterol-binding protein-like 3 (OSBPL3) in pancreatic ductal adenocarcinoma (PDAC) via cBioPortal and DAVID platform. (**A**) Bar plot of BIOCARTA-plentiful terms of OSBPL3 in PDAC patients. (**B**) Bar plot of KEGG-enriched terms of OSBPL3 in patients with PDAC.

**Figure 9:**
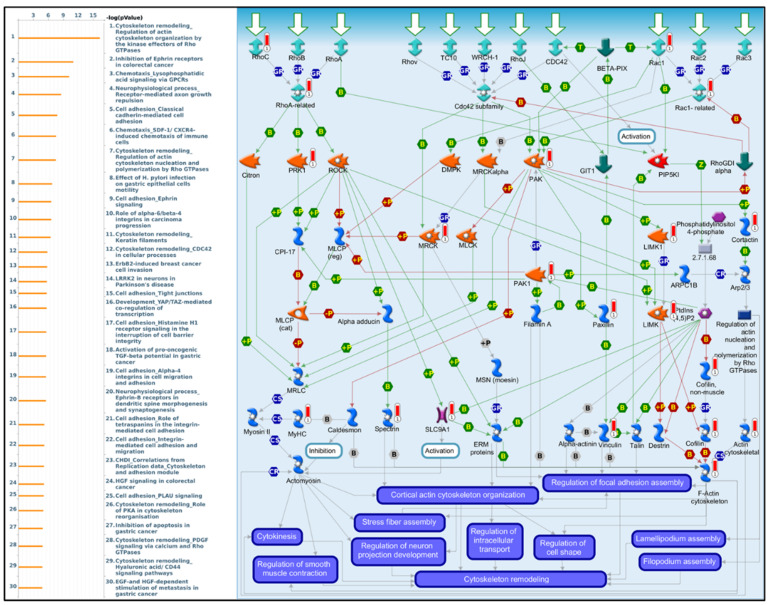
MetaCore pathway analysis of the co-expression gene network of oxysterol-binding protein-like 3 (OSBPL3) in pancreatic cancer patients. Downstream pathway analyses revealed that “Cytoskeleton remodeling_Regulation of actin cytoskeleton organization by the kinase effectors of Rho GTPases” might play an important role in pancreatic cancer development.

First, to explore the universally regulated pathways of all OSBPL members, we conducted a PPI evaluation of OSBPL members using the STRING database (Figure 7A). The sources of PPI networks of active interactions included test-mining, experiments, databases, co-expressions, neighborhoods, gene fusion, and co-occurrences, and so the diagram indicates nine members of the OSBPL family and their potential interacted proteins. Furthermore, through the KM plotter and a human protein atlas (HPA) analyses, we observed that the association between OSBPL3 expression in PDAC with poor prognosis and the immunohistochemistry also detects significant levels of OSBPL3 expression in PDAC However, regulation of the OSBPL3-related molecular mechanism remained less clear. Therefore, in order to comprehensively analyze the co-expression of OSBPL3, in this research, we downloaded the co-expression file and selected the first 1000 small-*p*-value data from cBioPortal before applying DAVID. In GOTERM MFs, there were correlated pathways, including ATP binding, integrin binding, receptor binding, etc., as shown in Figure 7B and Supplementary Table S3. Furthermore, we also analyzed the co-expressions of OSBPL3 from two different resources, BIOCARTA (Figure 8A) and KEGG (Figure 8B), and this was processed them by using DAVID analysis. In the BIOCARTA results, there were four genes (marked by red stars) in steps of the glycosylation of mammalian N-linked oligosaccharides (4%, *p* = 7.2× 10^−2^). In the KEGG diagram, there were 30 genes (3.1%, *p* = 5.1× 10^−6^) in the renin–angiotensin system (RAS) signaling pathway. In addition, the GeneGo MetaCore annotations of each BP suggested that the genes co-expressed with OSBPL3 were involved in cytoskeletal remodeling and ephrin-related pathways and networks such as “Cytoskeleton remodeling_Regulation of actin cytoskeleton organization by the kinase effectors of Rho GTPases”, “Inhibition of ephrin receptors in colorectal cancer”, and “Cell adhesion_Ephrin signaling”; thus, these played essential roles in pancreatic cancer (Figure 9; Supplementary Table S4). 

### 3.6. Associations of OSBPL Family Gene Transcriptional Levels and Immune-Infiltration in PDAC

**Figure 10:**
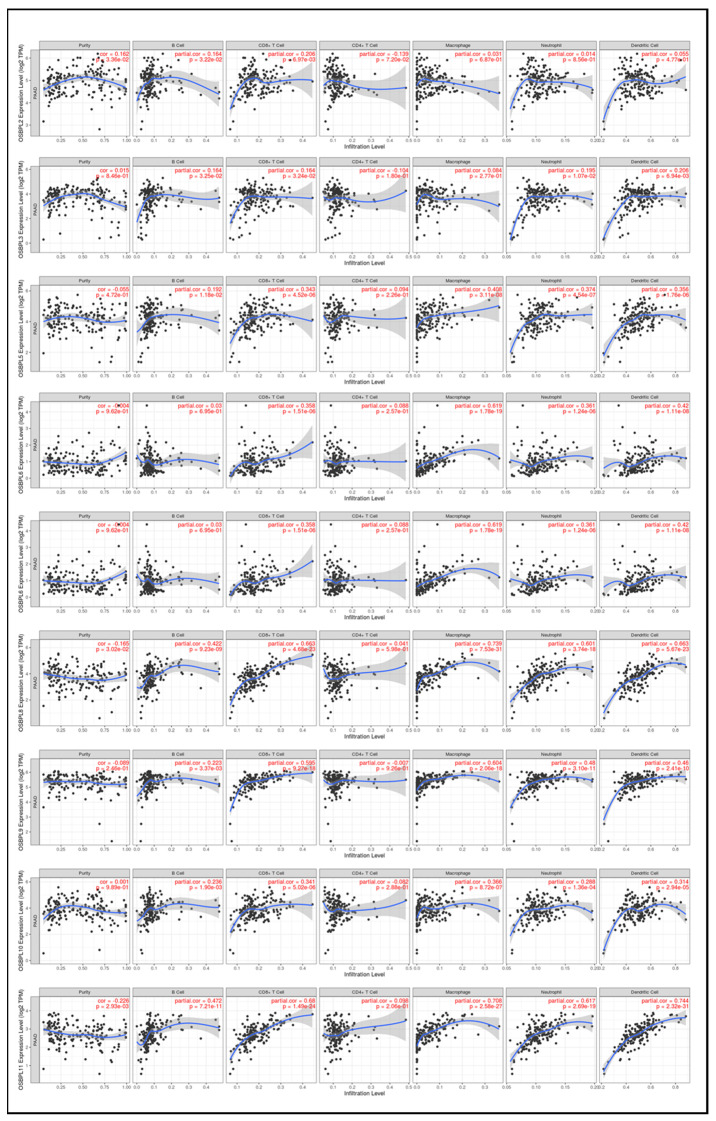
Association of expressions of oxysterol-binding protein-like (OSBPL) family members and tumor-infiltrating immune levels in pancreatic ductal adenocarcinoma (PDAC) using TIMER 2.0 data. The figures show the expression of each OSBPL family member with tumor purity and several tumor-infiltrating lymphocyte markers, such as B cell markers, CD8^+^ T cell markers, CD4 ^+^ T cell markers, macrophages, neutrophils, and dendritic cells. *p* < 0.05 was considered statistically significant.

Based on previous studies in immunotherapy, the tumor microenvironment (TME) of most cancers can be broadly identified as either tumor-infiltrating lymphocytes (TILs) (hot) or non-TILs (cold). Unfortunately, PDAC is a typical cold tumor [52]; thus, studies to detect further potential predictive biomarkers correlated with immunotherapeutic outcomes are necessary. Therefore, the TIMER database was used to explore the relationships between OSBPL members and immune cell infiltration (Figure 10) Results showed that the transcriptional levels of OSBPL2 were positively associated with the infiltration of B cells and CD4^+^ T cells (*p* < 0.05). OSBPL3 expression was positively associated with the infiltration of B cells, neutrophils, and DCs (*p* < 0.05). OSBPL5, OSBPL6, OSBPL8, OSBPL10, and OSBPL11 were positively associated with the infiltration of B cells, CD8+ T cells, macrophages, neutrophils, and DCs (*p* < 0.05). OSBPL7 was positively associated with the infiltration of B cells, CD8^+^ T cells, CD4^+^ T cells, and macrophages (*p* < 0.05). OSBPL9 was positively correlated with the infiltration of CD8^+^ T cells, macrophages, neutrophils, and DCs (*p* < 0.05). These results suggested that OSBPL genes may play critical roles in cancer immunology and could be biomarkers for immunotherapy.

**Figure 11:**
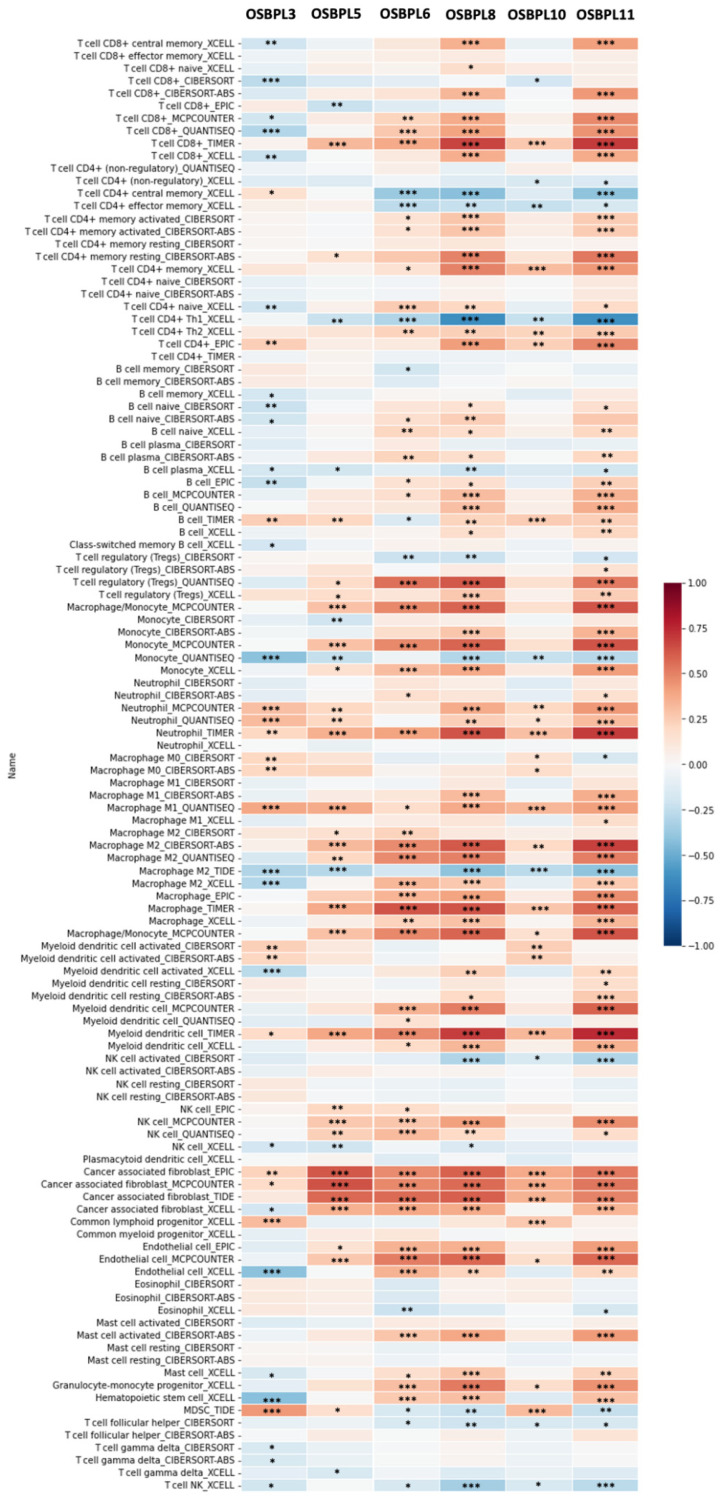
Heatmap of OSBPL3, OSBPL5, OSBPL6, OSBPL8, OSBPL10, and OSBPL11 expressions and immune infiltrates in pancreatic ductal adenocarcinoma (PDAC). The plot indicates correlations of PDAC, and the number of samples out of 116 immune infiltrates methods from six state-of-the-art algorithms, consisting of TIMER, EPIC, CIBERSORT, xCell, MCP-counter, and quantization. R-scores ranged −1.0−1.0. A value of r = 1 denotes a perfect positive correlation, while a value of r = −1 shows a perfect negative correlation. *: *p* < 0.05; **: *p* < 0.01; ***: *p* < 0.001.

Furthermore, when investigating relationships between immune cells and cancer cells in the TME, we noted that not only were cancer cells expressing OSBPL members, but furthermore that most immune cells invaded PDAC tumors and their subtypes with a high OSBPL expressions in multiple immunological de-convolution approaches. We further employed quantification algorithms (xCell, CIBERSORT, CIBERSORT abs.mode, EPIC, MCP-counter, TIMER, and quanTIseq) from TIMER to study relationships between OSBPL expressions and a comprehensive list of immune cells. As shown in Figure 11, OSBPL members exhibited the strongest positive correlations with the levels of CD4+ T cells, M1 macrophages, neutrophils, monocytes, and cancer-associated fibroblasts, while showing negative correlations with CD4^+^ T cells, type 2 helper T (Th2) cells, and monocytes by QuanTIseq. In particular, we utilized six- of the OSBPL gene family with the highest expressions, including OSBPL3, OSBPL5, OSBPL6, OSBPL8, OSBPL10, and OSBPL11, for further exploration. Among these genes, we observed that OSBPL6, OSBPL8, and OSBPL11 had strong interactions correlated with immune cell infiltration, suggesting that their important roles in immunological function and the TME.

## 4. Discussion

Pancreatic cancer, even resectable pancreatic cancer, has a very dismal prognosis despite advances in therapeutic modalities. Further understanding of the tumorigenesis process and identifying possible prognostic markers are crucial for developing therapeutic strategies. In previous studies, the *OSBPL* gene family was found to be a group of potential biomarkers for early cancer diagnosis. Moreover, in the mechanical regulation of OSBPL members, a recent study showed that GAB2 and GAB3, co-expressed with the OSBPL gene family were interrelated with much-shorter progression-free survival in ovarian cancer [53]. Among genes of this family, OSBPL3, OSPBL4, OSBPL5, and OSBL8 were reported to regulate or interact with other proteins involved in oncogenic signaling [54].

In this study, we demonstrated that the OSBPL3, OSBPL5, OSBPL8, OSBPL10, and OSBPL11 expression levels were significantly higher in PDAC. In particular, the OSBPL3, OSBPL5, and OSBPL6 expression levels were higher in stage IV PDAC. Furthermore, OSBPL3, OSBPL8, and OSBPL10 overexpression were associated with poor prognoses for PDAC patients and the co-expression analysis also showed several pathways related to tumorigenesis (Supplementary Tables S5 and S6). We also performed univariate and multivariate Cox regression analyses on OS which revealed the clinical impacts of OSBPL members on PDAC. As a result, we found that clinicopathological parameters and the value of OSBPL3 expression were significantly correlated with tumor stages in PDAC (Supplementary Tables S7 and S8). In addition, we demonstrated that high levels of gene amplification and mutations of OSBPL mambers were notable in PDAC. Furthermore, we analyzed genes co-expressed with OSBPL gene family members and showed that RAS signaling pathways were connected to cytoskeletal remodeling, endo-cytosis, adenosine monophosphate-activated kinase (AMPK) pathways, T-cell receptor signaling pathways, and the phosphatidylinositol 3-kinase (PI3K)-Akt signaling pathways which are a critical mechanistic pathways in OSBPL-expressing PDAC.

OSBPL3 is the most-studied gene family member and the main one to be associated with cancers. OSBPL3 overexpression was found to be involved in cell adhesion and interaction with R-Ras signaling, which promots tumor progression [2]. We used a PPI networks analysis to decipher possible biological functions of the candidate genes and disease progression. In our PPI networks and pathway analyses, we demonstrated the involvement of OSBPL3 with the ATP-binding, integrin-binding, and receptor-binding pathways. Meanwhile, from the BICARTA results, we found the co-expression of OSBPL3 with genes that regulate the glycosylation of mammalian N-linked oligosaccharides. Indeed, OSBPL3 could further regulate integrin function and is upregulated in pancreatic cancer tissues [55]. OSBPL5 was proved to be a poor prognostic marker among PDAC patients [56]. In addition, a previous study also showed that OSBPL5 interacts with the mammalian target of rapamycin (mTOR), and the PI3K/AKT/mTOR pathway is usually active in cancer [54]. Altogether, upregulation of these genes promotes tumor growth.

Furthermore, we analyzed genes co-expressed with of OSBPL3. We showed that the RAS signaling pathways that are connected to cytoskeletal remodeling, endocytosis, mitogen-activated protein kinase (MAPK) pathways, T-cell receptor signaling pathways, and PI3K-Akt signaling pathways, are play a critical mechanistic pathways in OSBPL-expressing PDAC. The RAS pathway was also reported to modulate tumor growth, angiogenesis, and tumor metastasis in pancreatic cancer [57]. Taken together, OSBPL3 might play a crucial role in tumorigenesis through regulating several critical signaling pathways.

In addition, previous studies show that PDAC is characterized as having a low tumor mutational burden (TMB)—defined as the total number of somatic mutations per coding area of a tumor genome—due to limited expressions of neoantigens, which activate T cells, in contrast to other solid tumors [58], thus leading to poor immune surveillance and poor responses to immunotherapy. Futhermore, tumor immune cell infiltration might serve as predictive markers for host immune responses to cancer [59]. Therefore, it is critical to identify the correlations between OSBPL members and immune infiltration, which requires further investigation for clinical applications. In our studies, we found that the expressions of OSBPL members were strongly related to various types of immune infiltrates. For example, several OSBPL members were positively correlated with the infiltration of B cells, T cells, macrophages, DCs, and neutrophils.

B cell subsets in PDAC were reported to upregulate immunosuppressive cytokines and inhibit T-cell-mediated tumor immunity [60]. The impact of the infiltrating of T cells on the TME is worth further investigation, and different subsets with distinct functions have been demonstrated. The loss of balance of T cell subsets might further facilitate tumorigenesis [61]. Furthermore, macrophages and neutrophils are crucial for the immunosuppressive TME and tumor progression [62]. Correlations between the transcription levels of *OSBPL* gene family members and immune cells clarified that OSBPLs members play significant roles in the immune control of PDAC. Taken together, OSBPL members could be biomarkers or novel therapeutic strategies for immunotherapy of PDAC.

## 5. Conclusions

In summary, by synthesizing diverse high-throughput databases, our research illustrates that OSBPL gene family members are potential therapeutic targets for PDAC and have great prognostic value. OSBPL3 and OSBPL8 were enhanced in PDAC patients and were able to forecast poor prognoses. Building on these results, we hope to provide fresh inspiration for developing therapies and clinical applications in the future.

## Data Availability

CBioPortal (https://cbioportal.org, accessed on 15 April 2021); The Human Protein Atlas (https://www.proteinatlas.org, accessed on 15 April 2021); Kaplan–Meier plot database (https://kmplot.com, accessed on 15 April 2021); MetaCore Analysis (https://portal.genego.com, accessed on 15 April 2021). The datasets used and/or analyzed during the current study are available from the corresponding author on reasonable request.

## Supplementary Materials

The following are available online at https://www.mdpi.com/article/10.3390/biomedicines9111601/s1, Table S1: Significant changes in expressions of oxysterol-binding protein-like (OSBPL) family members at the transcription level between different types of pancreatic ductal adenocarcinoma (PDAC) and normal pancreatic samples screened by Oncomine, Table S2: Associations of prognoses with transcriptional mRNA levels of oxysterol-binding protein-like (OSBPL) family members in patients with pancreatic ductal adenocarcinoma (PDAC), Table S3: The Gene Ontology (GO) function abundance research of oxysterol-binding protein (OSBP)-like (OSBPL)family and interrelated genes in pancreatic ductal adenocarcinoma (PDAC) using the cBioPortal and DAVID, Table S4: Pathway analysis of genes coexpressed with oxysterol-binding protein like-3 (OSBPL3) from public breast cancer databases using the MetaCore database (with *p* < 0.01 set as the cutoff value), Table S5: Pathway analysis of genes coexpressed with oxysterol-binding protein like-8 (OSBPL8) from public breast cancer databases using the MetaCore database (with *p* < 0.01 set as the cutoff value), Table S6: Pathway analysis of genes coexpressed with oxysterol-binding protein like-10 (OSBPL10) from public breast cancer databases using the MetaCore database (with *p* < 0.01 set as the cutoff value), Table S7: Univariate and multivariate Cox proportional hazards regression analysis of PDAC overall survival (OS) outcome. Factors showing significant relationship with OS from univariate analysis were then used for multivariate analysis from breast TCGA database. HR, hazard ratio; CI, confidence interval; *: *p* values < 0.05, Table S8: Multivariate analysis of OSBPL expression and relationships between it and clinicopathological parameters (age, treatment, stage, and TNM (tumor, node, metastasis) stage).
