# Supplementary material for: Potential Prognostic Biomarkers of OSBPL Family Genes in Patients with Pancreatic Ductal Adenocarcinoma
Source: Biomedicines. 2021 Nov 3;9(11):1601. doi: 10.3390/biomedicines9111601 (PMC8615799; doi:10.3390/biomedicines9111601)

SUPPLEMENTARY MATERIAL:

Table S1 Significant changes in expressions of oxysterol-binding protein-like (OSBPL) family members at the transcription level between different types of pancreatic ductal adenocarcinoma (PDAC) and normal pancreatic samples screened by Oncomine

	Type of PAAD *vs.* normal	Multiple of change	*t*-test	*p* value	Ref.
OSBPL2	NA	NA	NA	NA	NA
OSBPL3	Pancreatic carcinoma	2.872	5.595	9.07E-5	[19]
	Pancreatic carcinoma	3.571	6.017	2.31E-6	[20]
	PDAC	3.221	7.475	3.30E-10	[21]
	Pancreatic adenocarcinoma	1.952	3.239	0.003	[22]
OSBPL5	PDAC	-1.636	-2.964	0.008	[23]
OSBPL6	Pancreatic adenocarcinoma	-6.521	-4.006	0.002	[24]
OSBPL7	NA	NA	NA	NA	NA
OSPBL8	Pancreatic carcinoma	3.000	3.437	0.002	[19]
	PDAC	2.119	6.366	3.97E-8	[23]
OSPBL9	NA	NA	NA	NA	NA
OSBPL10	Pancreatic carcinoma	3.438	5.770	7.16E-5	[19]
	Pancreatic adenocarcinoma	4.158	5.531	5.45E-4	[22]
	PDAC	4.814	7.754	1.95E-10	[21]
	Pancreatic carcinoma	3.527	4.901	5.31E-5	[20]
OSBPL11	Pancreatic carcinoma	1.509	3.329	0.002	[19]

NA, not available; PDAC, pancreatic ductal adenocarcinoma

Table S2 Associations of prognoses with transcription mRNA levels of oxysterol-binding protein-like (OSBPL) family members in patients with pancreatic ductal adenocarcinoma (PDAC)

	**Kaplan-Meier plotter (Logrank *p*); HR**			
	**RFS**		**OS**	
OSBPL2	0.39;	0.7	0.22;	0.78
OSBPL3	0.36;	1.47	0.0072;	1.76
OSBPL5	0.097;	2.02	0.97;	0.99
OSBPL6	0.82;	0.91	0.021;	0.61
OSBPL7	0.21;	1.7	0.56;	1.13
OSBPL8	0.021;	2.83	0.41;	1.19
OSBPL9	0.36;	1.48	0.32;	1.23
OSBPL10	0.0031;	3.77	0.0089;	1.73
OSBPL11	0.016;	2.94	0.69;	1.09

HR, hazard ratio; RFS, recurrence-free survival; OS, overall survival.

Table S3 The Gene Ontology (GO) function abundance research of oxysterol-binding protein (OSBP)-like (OSBPL)family and interrelated genes in pancreatic ductal adenocarcinoma (PDAC) using the cBioPortal and DAVID

**GO**	**Category**	**Description**	**Count**	**%**	**Log10(*p*)**	**Log10(q)**
**Molecular function**	GO molecular function	actin binding	33	3.4	1.3E-5	1.2E-2
**Molecular function**	GO molecular function	calcium ion binding	62	6.3	4.8E-5	2.3E-2
**Molecular function**	GO molecular function	SH3 domain binding	18	1.8	1.0E-4	2.8E-2
**Molecular function**	GO molecular function	inositol 1,4,5 trisphosphate binding	6	0.6	1.2E-4	2.8E-2
**Molecular function**	GO molecular function	ephrin receptor binding	8	0.8	2.3E-4	4.5E-2
**Molecular function**	GO molecular function	heparin binding	20	2.0	4.7E-4	7.6E-2
**Molecular function**	GO molecular function	growth factor binding	7	0.7	2.0E-3	2.6E-1
**Molecular function**	GO molecular function	transmembrane receptor protein tyrosine kinase activity	8	0.8	2.6E-3	2.6E-1
**Molecular function**	GO molecular function	protein kinase activity	32	3.3	2.7E-3	2.6E-1
**Molecular function**	GO molecular function	actin filament binding	16	1.6	2.8E-3	2.6E-1
**Molecular function**	GO molecular function	protein tyrosine kinase activity	16	1.6	3.0E-3	2.6E-1
**Molecular function**	GO molecular function	vascular endothelial growth factor-activated receptor activity	4	0.4	3.9E-3	3.1E-1
**Molecular function**	GO molecular function	protein binding	482	49.2	5.6E-3	3.9E-1
**Molecular function**	GO molecular function	3',5'-cyclic-AMP phosphodiesterase activity	5	0.5	5.7E-3	3.9E-1
**Molecular function**	GO molecular function	SH3/SH2 adaptor activity	9	0.9	6.8E-3	4.3E-1
**Molecular**	GO molecular	receptor binding	30	3.1	7.2E-3	4.3E-1

**function**	function					
**Molecular function**	GO molecular function	non-membrane spanning protein tyrosine kinase activity	8	0.8	7.9E-3	4.4E-1
**Molecular function**	GO molecular function	profilin binding	4	0.4	8.6E-3	4.4E-1
**Molecular function**	GO molecular function	transmembrane-ephrin receptor activity	4	0.4	8.6E-3	4.4E-1
**Molecular function**	GO molecular function	ATPase binding	10	1.0	1.2E-2	5.7E-1
**Molecular function**	GO molecular function	cell adhesion molecule binding	9	0.9	1.2E-2	5.7E-1
**Molecular function**	GO molecular function	protein phosphatase binding	9	0.9	1.4E-2	6.0E-1
**Molecular function**	GO molecular function	Rho guanyl-nucleotide exchange factor activity	10	1.0	1.5E-2	6.2E-1
**Molecular function**	GO molecular function	calmodulin binding	18	1.8	1.6E-2	6.2E-1
**Molecular function**	GO molecular function	integrin binding	12	1.2	1.7E-2	6.6E-1
**Molecular function**	GO molecular function	guanylate kinase activity	4	0.4	2.0E-2	7.2E-1
**Molecular function**	GO molecular function	store-operated calcium channel activity	4	0.4	2.0E-2	7.2E-1
**Molecular function**	GO molecular function	vascular endothelial growth factor binding	3	0.3	2.3E-2	7.9E-1
**Molecular function**	GO molecular function	3',5'-cyclic-nucleotide phosphodiesterase activity	5	0.5	2.7E-2	8.3E-1
**Molecular function**	GO molecular function	GTPase activator activity	23	2.3	2.7E-2	8.3E-1
**Molecular function**	GO molecular function	ion channel binding	12	1.2	2.8E-2	8.3E-1
**Molecular function**	GO molecular function	identical protein binding	51	5.2	2.8E-2	8.3E-1
**Molecular function**	GO molecular function	PDZ domain binding	10	1.0	3.0E-2	8.3E-1
**Molecular function**	GO molecular function	RNA polymerase II transcription factor binding	7	0.7	3.0E-2	8.3E-1

**Molecular function**	GO molecular function	serine-type carboxypeptidase activity	4	0.4	3.1E-2	8.3E-1
**Molecular function**	GO molecular function	collagen binding	8	0.8	3.1E-2	8.3E-1
**Molecular function**	GO molecular function	alpha-N-acetylgalactosaminide alpha-2,6-sialyltransferase activity	3	0.3	3.3E-2	8.7E-1
**Molecular function**	GO molecular function	kinase binding	9	0.9	3.5E-2	8.8E-1
**Molecular function**	GO molecular function	phosphatidylinositol-4,5-bisphosphate 3-kinase activity	8	0.8	3.6E-2	8.8E-1
**Molecular function**	GO molecular function	guanyl-nucleotide exchange factor activity	12	1.2	3.7E-2	8.8E-1
**Molecular function**	GO molecular function	serine-type peptidase activity	8	0.8	3.9E-2	9.2E-1
**Molecular function**	GO molecular function	fibronectin binding	5	0.5	4.0E-2	9.2E-1
**Molecular function**	GO molecular function	Rac GTPase binding	6	0.6	5.0E-2	1.0E-0
**Molecular function**	GO molecular function	RNA polymerase II transcription factor activity, sequence-specific DNA binding	15	1.5	5.2E-2	1.0E-0
**Molecular function**	GO molecular function	transcriptional activator activity, RNA polymerase II core promoter proximal region sequence-specific binding	19	1.9	5.5E-2	1.0E-0
**Molecular function**	GO molecular function	signal transducer activity	17	1.7	5.5E-2	1.0E-0
**Molecular function**	GO molecular function	protein homodimerization activity	48	4.9	5.5E-2	1.0E-0
**Molecular function**	GO molecular function	patched binding	3	0.3	5.8E-2	1.0E-0
**Molecular function**	GO molecular function	sphingosine-1-phosphate receptor activity	3	0.3	5.8E-2	1.0E-0

**Molecular function**	GO molecular function	tubulin binding	6	0.6	5.9E-2	1.0E-0
**Molecular function**	GO molecular function	protein phosphatase 1 binding	4	0.4	6.0E-2	1.0E-0
**Molecular function**	GO molecular function	G-protein coupled peptide receptor activity	4	0.4	6.0E-2	1.0E-0
**Molecular function**	GO molecular function	coreceptor activity	5	0.5	6.3E-2	1.0E-0
**Molecular function**	GO molecular function	sialyltransferase activity	4	0.4	6.8E-2	1.0E-0
**Molecular function**	GO molecular function	insulin receptor binding	5	0.5	6.9E-2	1.0E-0
**Molecular function**	GO molecular function	epidermal growth factor receptor binding	5	0.5	6.9E-2	1.0E-0
**Molecular function**	GO molecular function	fucosyltransferase activity	3	0.3	7.3E-2	1.0E-0
**Molecular function**	GO molecular function	ATP binding	89	9.1	7.3E-2	1.0E-0
**Molecular function**	GO molecular function	transcriptional repressor activity, RNA polymerase II transcription regulatory region sequence-specific binding	7	0.7	7.7E-2	1.0E-0
**Molecular function**	GO molecular function	structural molecule activity	19	1.9	7.8E-2	1.0E-0
**Molecular function**	GO molecular function	protein C-terminus binding	15	1.5	7.8E-2	1.0E-0
**Molecular function**	GO molecular function	lipid binding	13	1.3	8.1E-2	1.0E-0
**Molecular function**	GO molecular function	GTP binding	27	2.8	8.2E-2	1.0E-0
**Molecular function**	GO molecular function	glutathione peroxidase activity	4	0.4	8.7E-2	1.0E-0
**Molecular function**	GO molecular function	actinin binding	3	0.3	8.8E-2	1.0E-0
**Molecular function**	GO molecular function	protein phosphatase type 1 regulator activity	3	0.3	8.8E-2	1.0E-0
**Molecular**	GO molecular	receptor signaling protein	3	0.3	8.8E-2	1.0E-0

**function**	function	tyrosine kinase activity				
**Molecular function**	GO molecular function	phosphoprotein binding	5	0.5	9.1E-2	1.0E-0
**Molecular function**	GO molecular function	scaffold protein binding	6	0.6	9.4E-2	1.0E-0
**Molecular function**	GO molecular function	activating transcription factor binding	4	0.4	9.7E-2	1.0E-0
**Molecular function**	GO molecular function	gap junction channel activity involved in SA node cell-atrial cardiac muscle cell electrical coupling	2	0.2	9.9E-2	1.0E-0
**Molecular function**	GO molecular function	chemokine (C-C motif) ligand 5 binding	2	0.2	9.9E-2	1.0E-0
**Molecular function**	GO molecular function	chondroitin-glucuronate 5-epimerase activity	2	0.2	9.9E-2	1.0E-0
**Molecular function**	GO molecular function	calcium- and calmodulin-regulated 3',5'-cyclic-GMP phosphodiesterase activity	2	0.2	9.9E-2	1.0E-0
**Molecular function**	GO molecular function	sphingolipid delta-4 desaturase activity	2	0.2	9.9E-2	1.0E-0

Table S4: Pathway analysis of genes coexpressed with oxysterol-binding protein like- 3 (OSBPL3) from public breast cancer databases using the MetaCore database (with $p<0.01$ set as the cutoff value)

#	Map	*p* Value	Network objects from active data
**1**	Cytoskeleton remodeling_Regulation of actin cytoskeleton organization by the kinase effectors of Rho GTPases	5.09E-17	RhoC, Vinculin, RhoA-related, LIMK1, Cortactin, MyHC, Actin cytoskeletal, LIMK, PAK, Cofilin, non-muscle, F-Actin cytoskeleton, Spectrin, SLC9A1, Paxillin, Cofilin, Rac1-related, Rac1, MRCK, PAK1, PRK1
**2**	Inhibition of Ephrin receptors in colorectal cancer	1.31E-11	Ephrin-A, Ephrin-B1, Ephrin-A receptor 1, KLF5, Ephrin-B2, Ephrin-B, WNT, Ephrin-A receptors, Ephrin-A receptor 2, SMAD3, Paxillin, Rac1
**3**	Chemotaxis_Lysophosphatidic acid signaling via GPCRs	8.84E-11	LPAR2, PKC-zeta, Bcl-XL, Vinculin, p130CAS, DIA1, Rho GTPase, PLC-beta3, Actin cytoskeletal, LIMK, PAK, HDAC7, F-Actin cytoskeleton, Paxillin, Cofilin, Rac1, ERK1/2, PKC, YAP1 (YAp65), PRK1, PLC-beta
**4**	Neurophysiological process_Receptor-mediated axon growth repulsion	3.89E-09	Ephrin-A, LIMK1, Cortactin, Plexin A1, Actin cytoskeletal, Fyn, Ephrin-A receptors, Ephrin-A receptor 2, F-Actin cytoskeleton, Cofilin, Rac1, PAK1
**5**	Cell adhesion_Classical cadherin-mediated cell adhesion	2.29E-08	Vinculin, Cortactin, Formin, p120-catenin, Actin cytoskeletal, F-Actin cytoskeleton, Rac1, Plakoglobin, F-Actin
**6**	Chemotaxis_SDF-1/ CXCR4-induced chemotaxis of immune cells	4.21E-08	PKC-zeta, Vinculin, p130CAS, LIMK1, SFK, Fyn, PAK, F-Actin cytoskeleton, Paxillin, Cofilin, Rac1, ERK1/2, PAK1, PLC-beta
**7**	Cytoskeleton remodeling_Regulation of actin cytoskeleton nucleation and polymerization by Rho GTPases	4.72E-08	RhoC, RhoA-related, RhoD, DIA1, DRF, Actin cytoskeletal, RhoF (Rif), F-Actin cytoskeleton, mDIA2(DIAPH3), Rac1-related, Rac1
**8**	Effect of H. pylori infection on gastric epithelial cells motility	2.59E-07	Vinculin, Cortactin, p120-catenin, Actin cytoskeletal, Actin, MARK2, Paxillin, Rac1, ERK1/2, PAK1
**9**	Cell adhesion_Ephrin signaling	4.09E-07	Ephrin-A, ADAM10, Ephrin-A5, Fyn, Ephrin-B, Ephrin-A receptors, Ephrin-A receptor 2, Paxillin, Rac1, PAK1

**10**	Role of alpha-6/beta-4 integrins in carcinoma progression	4.09E-07	Plectin 1, ITGB4, LIMK1, HGF receptor (Met), Actin cytoskeletal, alpha-6/beta-4 integrin, MSP receptor (RON), Cofilin, Rac1, PAK1
**11**	Cytoskeleton remodeling_Keratin filaments	5.35E-07	Plectin 1, Keratin 16, PPL(periplakin), Envoplakin, Actin cytoskeletal, Keratin 7, Keratin 6A, Keratin 19, Tubulin alpha
**12**	Cytoskeleton remodeling_CDC42 in cellular processes	2.38E-06	PKC-zeta, LIMK1, Actin cytoskeletal, PARD6, F-Actin cytoskeleton, Cofilin, PAK1
**13**	ErbB2-induced breast cancer cell invasion	2.66E-06	Calpain 1(mu), PLAUR (uPAR), p120-catenin, Actin cytoskeletal, SLK, Cofilin, non-muscle, PLAU (UPA), Cofilin, Rac1, ERK1/2, PAK1
**14**	LRRK2 in neurons in Parkinson's disease	3.00E-06	PKC-zeta, Actin cytoskeletal, MARK2, ACTB, Rac1, ERK1/2, PAK1, 14-3-3
**15**	Cell adhesion_Tight junctions	3.30E-06	PKC-zeta, Cortactin, ZO-3, Actin cytoskeletal, PARD6, Actin, p114-RhoGEF, Tubulin alpha, F-Actin
**16**	Development_YAP/TAZ-mediated co-regulation of transcription	3.46E-06	Bcl-XL, Oct-3/4, ID1, TEF-5, KLF5, UCA1, SMAD3, PML, YAP1 (YAp65), CDK6
**17**	Cell adhesion_Histamine H1 receptor signaling in the interruption of cell barrier integrity	4.02E-06	Histamine H1 receptor, Vinculin, p130CAS, LIMK1, p120-catenin, Actin cytoskeletal, Paxillin, Cofilin, PLC-beta
**18**	Activation of pro-oncogenic TGF-beta potential in gastric cancer	4.45E-06	GSTP1, ITGA3, p130CAS, Cofilin, non-muscle, SMAD3, Paxillin, ITGA2
**19**	Cell adhesion_Alpha-4 integrins in cell migration and adhesion	4.83E-06	p130CAS, LIMK1, Actin cytoskeletal, F-Actin cytoskeleton, Paxillin, Cofilin, Rac1, PAK1
**20**	Neurophysiological process_Ephrin-B receptors in dendritic spine morphogenesis and synaptogenesis	4.83E-06	Dynamin-2, Cortactin, Actin cytoskeletal, Fyn, Ephrin-B, Synbindin, Rac1, PAK1
**21**	Cell adhesion_Role of tetraspanins in the integrin-mediated cell adhesion	1.00E-05	Plectin 1, ITGB4, ITGA3, p130CAS, Actin cytoskeletal, CD82, ITGA6, alpha-6/beta-4 integrin, Paxillin
**22**	Cell adhesion_Integrin-mediated cell adhesion and migration	1.04E-05	PKC-zeta, Vinculin, p130CAS, Actin cytoskeletal, PARD6, F-Actin cytoskeleton, Paxillin, Rac1, PKC, PAK1

**23**	CHDI_Correlations from Replication data_Cytoskeleton and adhesion module	1.20E-05	Vinculin, PLAUR (uPAR), MyHC, Actin cytoskeletal, Fyn, Ephrin-B, PLAU (UPA), Paxillin, Rac1, PAK1
**24**	HGF signaling in colorectal cancer	1.20E-05	HGF receptor (Met), ADAM10, PLAUR (uPAR), Actin cytoskeletal, MACC1, HAI-1, LAMA3 (Epiligrin), Rac1, ERK1/2, LAMB3
**25**	Cell adhesion_PLAU signaling	1.38E-05	Casein kinase II, alpha chains, p130CAS, PLAUR (uPAR), sUPAR, F-Actin cytoskeleton, PLAU (UPA), Paxillin, Rac1, ERK1/2, PAK1
**26**	Cytoskeleton remodeling_Role of PKA in cytoskeleton reorganization	1.69E-05	LIMK1, PLC-beta3, Actin cytoskeletal, F-Actin cytoskeleton, Paxillin, Cofilin, Rac1, PAK1
**27**	Inhibition of apoptosis in gastric cancer	2.04E-05	FADD, Bcl-XL, HGF receptor (Met), c-FLIP(Short), tBid, XAF1, PAK1, Bid
**28**	Cytoskeleton remodeling_PDGF signaling via calcium and Rho GTPases	2.22E-05	Vinculin, p130CAS, Dynamin-2, Cortactin, Actin cytoskeletal, Fyn, Paxillin, Rac1, PKC, PAK1, F-Actin
**29**	Cytoskeleton remodeling_Hyaluronic acid/ CD44 signaling pathways	2.44E-05	Dynamin-2, HGF receptor (Met), Cortactin, Actin cytoskeletal, Fyn, Actin, Rac1, ERK1/2
**30**	EGF- and HGF-dependent stimulation of metastasis in gastric cancer	2.61E-05	ITGB4, HGF receptor (Met), alpha-6/beta-4 integrin, PLAU (UPA), Paxillin, ERK1/2

Table S5: Pathway analysis of genes coexpressed with oxysterol-binding protein like-8 (OSBPL8) from public breast cancer databases using the MetaCore database (with $p<0.01$ set as the cutoff value)

#	Map	*p* Value	Network objects from active data
**1**	TGF-beta-induced fibroblast/ myofibroblast migration and extracellular matrix production in asthmatic airways	1.13E-15	ITGA1, TIMP2, SMAD2, TGF-beta 3, Fibronectin, ITGB1, HAS2, PI3K cat class IA, PAI1, COL4A1, TAK1(MAP3K7), COL1A2, TIMP3, ERK1/2, AKT(PKB), MMP-2, Collagen III, Collagen IV, Thrombospondin 2, Decorin
**2**	IL-1 beta- and Endothelin-1-induced fibroblast/ myofibroblast migration and extracellular matrix production in asthmatic airways	1.36E-15	IL-1RI, Thrombospondin 1, Fibronectin, HAS2, PAI1, COL4A1, EDNRB, COL1A2, EDNRA, TIMP3, CTGF, ERK1/2, PDGF-R-beta, MMP-2, Versican, Collagen III, Decorin

**3**	Cell adhesion_ECM remodeling	1.16E-10	TIMP2, MSN (moesin), Fibronectin, Nidogen, Osteonectin, MMP-16, LAMA4, PAI1, alpha-1/beta-1 integrin, Syndecan-2, TIMP3, MMP-2, Versican, Collagen III, Collagen IV
**4**	Extracellular matrix-regulated proliferation of airway smooth muscle cells in asthma	4.45E-10	alpha-4/beta-1 integrin, DAB2, SMAD2, Fibronectin, ATF-2, PI3K cat class IA, TAK1(MAP3K7), CTGF, ERK1/2, Collagen III, Collagen IV, Decorin
**5**	Chemotaxis_Lysophosphatidic acid signaling via GPCRs	8.14E-10	LPAR1, CREB1, FKHR, MLCP (reg), Vinculin, ATF-2, G-protein beta/gamma, Tcf(Lef), Rho GTPase, HAS2, PRKD1, TAZ, G-protein alpha-12 family, ADAM17, GSK3 beta, PAK, Cofilin, CTGF, ERK1/2, YAP1 (YAp65), AKT(PKB)
**6**	Development_Regulation of epithelial-to-mesenchymal transition (EMT)	1.19E-09	IL-1RI, CREB1, SMAD2, TGF-beta 3, Fibronectin, ATF-2, Caldesmon, TCF8, SLUG, PAI1, Lef-1, EDNRA, SIP1 (ZFHX1B), PDGF-R-beta, MMP-2
**7**	Development_TGF-beta-dependent induction of EMT via SMADs	6.93E-09	SMAD2, TGF-beta 3, Fibronectin, TCF8, TGF-beta, ETS1, SLUG, PAI1, Lef-1, SIP1 (ZFHX1B), MMP-2
**8**	Glucocorticoid-induced elevation of intraocular pressure as glaucoma risk factor	7.22E-09	TRIO, GCR, SENP1, Thrombospondin 1, Fibronectin, ITGB1, PI3K cat class IA, GCR Alpha, PAI1, COL4A1, GCR Beta, MLCK, MMP-2, Collagen IV
**9**	Role of TGF-beta 1 in fibrosis development after myocardial infarction	1.83E-08	TIMP2, SMAD2, Thrombospondin 1, Fibronectin, PAI1, EDNRB, COL1A2, EDNRA, CTGF, MMP-2, Collagen III
**10**	Development_TGF-beta-dependent induction of EMT via MAPK	1.97E-08	PTEN, DAB2, TGF-beta 3, Fibronectin, ATF-2, ITGB1, TGF-beta, alpha-V/beta-1 integrin, PAI1, TAK1(MAP3K7), ERK1/2, MMP-2
**11**	Chemotaxis_SDF-1/ CXCR4-induced chemotaxis of immune cells	2.59E-08	alpha-4/beta-1 integrin, Talin, Vinculin, ITGB1, G-protein beta/gamma, PKA-reg (cAMP-dependent), PI3K cat class IA, Btk, PAK, PAK2, CD45, Cofilin, ERK1/2, G-protein alpha-13, AKT(PKB)

**12**	Cytoskeleton remodeling_Regulation of actin cytoskeleton organization by the kinase effectors of Rho GTPases	2.76E-08	RhoJ, Talin, MLCP (reg), Vinculin, MSN (moesin), Caldesmon, ERM proteins, TC10, MyHC, PAK, MLCK, Cofilin, Cdc42 subfamily
**13**	Development_SLIT-ROBO1 signaling	3.32E-08	SLIT3, ROBO1, PI3K cat class IA, SSH1L, Calcineurin A (catalytic), Cytohesin3, PAK2, SLIT2, Cofilin, AKT(PKB), FLII
**14**	TGF-beta 1-induced transactivation of membrane receptors signaling in HCC	4.18E-08	PDGF receptor, PTEN, Fibronectin, ITGB1, TGF-beta, PI3K cat class IA, SLUG, GSK3 beta, Lef-1, Cofilin, PDGF-R-beta, AKT(PKB)
**15**	Development_Stimulation of differentiation of mouse embryonic fibroblasts into adipocytes by extracellular factors	4.59E-08	CREB1, FKHR, ATF-2, EGR2 (Krox20), SHP-2, PI3K cat class IA (p110-alpha), PKA-reg (cAMP-dependent), HIVEP2, ERK1 (MAPK3), ATF-1, ERK1/2, Lysyl oxidase, BMP receptor 2, ERK2 (MAPK1)
**16**	Signal transduction_Angiotensin II/ AGTR1 signaling via p38, ERK and PI3K	1.11E-07	PDGF-C, CREB1, FKHR, WISP1, G-protein beta/gamma, ETS1, ADAM17, PI3K cat class IA, RECK, PAI1, ERK1/2, MEF2C, PDGF-R-beta, SP3, AKT(PKB), MMP-2
**17**	Signal transduction_Angiotensin II/AGTR1 signaling via Notch, Beta-catenin and NF-κB pathways	1.13E-07	Fibronectin, WISP1, PRKD1, ADAM17, GSK3 beta, TAK1(MAP3K7), TCF7L2 (TCF4), Connexin 43, CTGF, ERK1/2, YAP1 (YAp65), ERK2 (MAPK1), AKT(PKB), MMP-2
**18**	Ovarian cancer (main signaling cascades)	1.17E-07	LPAR1, PTEN, CREB1, G-protein beta/gamma, Tcf(Lef), PI3K cat class IA (p110-alpha), PKA-reg (cAMP-dependent), PI3K cat class IA, GSK3 beta, EDNRA, ERK1/2, AKT(PKB), MMP-2
**19**	TGF-beta signaling via kinase cascades in breast cancer	2.44E-07	ADAM12, TIMP2, ATF-2, ITGB1, ADAM17, PAI1, TAK1(MAP3K7), ERK1/2, TAB2, ITGAV, AKT(PKB), MMP-2
**20**	Stimulation of TGF-beta signaling in lung cancer	2.57E-07	Vinculin, SMAD2, TGF-beta 3, Fibronectin, ITGB1, TGF-beta, PI3K cat class IA (p110-alpha), SLUG, PAI1, AKT(PKB), MMP-2

**21**	Stellate cells activation and liver fibrosis	2.92E-07	PDGF receptor, IL-1RI, DAB2, SMAD2, Tcf(Lef), PI3K cat class IA, ERK1 (MAPK3), GSK3 beta, COL1A2, ERK2 (MAPK1), PDGF-R-beta, AKT(PKB), MMP-2
**22**	Role of alpha-V/ beta-6 integrin in colorectal cancer	3.43E-07	Fibronectin, LTBP1, ETS1, ERK1/2, ITGAV, ERK2 (MAPK1), MMP-2, Collagen IV
**23**	Cell adhesion_Integrin inside-out signaling	3.61E-07	Talin, Vinculin, Fibronectin, ITGB1, G-protein beta/gamma, G-protein alpha-12 family, ERK1 (MAPK3), alpha-1/beta-1 integrin, PAR1, Cytohesin3, ERK1/2, ERK2 (MAPK1)
**24**	Role of stellate cells in progression of pancreatic cancer	3.61E-07	PDGF receptor, SMAD2, Fibronectin, PI3K cat class IA, RECK, COL1A2, CTGF, ERK1/2, PDGF-R-beta, AKT(PKB), MMP-2, Collagen III
**25**	Fibroblast differentiation to myofibroblasts in asthmatic airways	3.63E-07	SMAD2, Fibronectin, ITGB1, PI3K cat class IA, TAK1(MAP3K7), EDNRB, EDNRA, ERK1/2, AKT(PKB)
**26**	IGF-1 signaling in multiple myeloma	4.02E-07	GSK3 alpha/beta, PTEN, FKHR, Fibronectin, ITGB1, PRKD1, PI3K cat class IA, ERK1 (MAPK3), ERK1/2, ERK2 (MAPK1), AKT(PKB)
**27**	Development_Negative regulation of STK3/4 (Hippo) pathway and positive regulation of YAP/TAZ function	5.25E-07	LPAR1, LATS2, PJA2, MOBKL1A, MLCP (reg), TAZ, G-protein alpha-12 family, ERK1 (MAPK3), PAR1, Itch, ERK1/2, YAP1 (YAp65)
**28**	Development_Role of PKR1 and ILK in cardiac progenitor cells	6.55E-07	alpha-4/beta-1 integrin, Fibronectin, ITGB1, Tcf(Lef), PI3K cat class IA, GSK3 beta, Lef-1, ERK1/2, AKT(PKB)
**29**	G protein-coupled receptors signaling in lung cancer	7.89E-07	LPAR1, PGE2R3, G-protein beta/gamma, Galpha(i)-specific peptide GPCRs, G-protein alpha-12 family, PKA-reg (cAMP-dependent), ADAM17, Galpha(q)-specific peptide GPCRs, EDNRB, EDNRA, ERK1/2, AKT(PKB), MMP-2

**30**	Apoptosis and survival_NGF/ TrkA PI3K-mediated signaling	9.22E-07	TRIO, CREB1, FKHR, MLCP (reg), MSN (moesin), PI3K cat class IA, SSH1L, GSK3 beta, Calcineurin A (catalytic), PAK2, Cofilin, ERK1/2, AKT(PKB)

Table S6: Pathway analysis of genes coexpressed with oxysterol-binding protein like-10 (OSBPL10) from public breast cancer databases using the MetaCore database (with $p<0.01$ set as the cutoff value)

#	Map	*p* Value	Network objects from active data
**1**	Ligand-independent activation of Androgen receptor in Prostate Cancer	1.76E-09	K-RAS, Bcl-XL, PDK (PDPK1), Beta-catenin, IRS-1, Tcf(Lef), PP2A regulatory, PI3K cat class IA, c-Raf-1, GSK3 beta, IBP3, Kallikrein 3 (PSA)
**2**	Stem cells_Cooperation between Hedgehog, IGF-2 and HGF signaling pathways in medulloblastoma stem cells	2.82E-09	Bcl-XL, PDK (PDPK1), HGF receptor (Met), Beta-catenin, IRS-1, Tcf(Lef), PI3K cat class IA, GSK3 beta, Lef-1
**3**	Canonical Leptin pathways in breast cancer	7.11E-09	PDK (PDPK1), Beta-catenin, IRS-1, Tcf(Lef), PI3K cat class IA, c-Raf-1, GSK3 beta, RelA (p65 NF-kB subunit), NF-kB, SP1
**4**	IGF family signaling in colorectal cancer	8.47E-08	GSK3 alpha/beta, Bcl-XL, Beta-catenin, IRS-1, c-Raf-1, GSK3 beta, RelA (p65 NF-kB subunit), IBP, NF-kB, IBP3
**5**	E-cadherin signaling and its regulation in gastric cancer	1.66E-07	RhoA, HGF receptor (Met), Beta-catenin, p120-catenin, WNT, Alpha-actinin, GSK3 beta, Plakoglobin
**6**	Development_Growth factors in regulation of oligodendrocyte precursor cell survival	2.09E-07	Bcl-XL, PDK (PDPK1), IRS-1, Fyn, PI3K cat class IA, c-Raf-1, GSK3 beta, RelA (p65 NF-kB subunit)
**7**	Canonical WNT signaling pathway in colorectal cancer	2.16E-07	K-RAS, Galectin-3, HGF receptor (Met), Beta-catenin, WNT, BMP4, PI3K cat class IA, GSK3 beta, Lef-1, WNT2
**8**	IGF signaling in HCC	2.72E-07	PDK (PDPK1), HGF receptor (Met), IRS-1, PI3K cat class IA, c-Raf-1, GSK3 beta, PKC, IBP3, SP1
**9**	Development_Positive regulation of WNT/Beta-catenin signaling in the nucleus	3.32E-07	FHL2, SOX11, Beta-catenin, Tcf(Lef), RUNX, WNT, GSK3 beta, Lef-1, FOXP1, YAP1 (YAp65)
**10**	Signal transduction_PDGF signaling via PI3K/AKT and NF-κB pathways	3.82E-07	K-RAS, PDK1, PDK (PDPK1), HXK2, Beta-catenin, PI3K cat class IA (p110-beta), GSK3 beta, RelA (p65 NF-kB subunit), NF-kB, SP1
**11**	Chemotaxis_Lysophosphatidic acid signaling via GPCRs	4.47E-07	RhoA, Bcl-XL, Vinculin, PDK (PDPK1), Beta-catenin, PI3K cat class IA (p110-beta), Tcf(Lef), Rho GTPase, TAZ, c-Raf-1, GSK3 beta, PKC, YAP1 (YAp65)
**12**	Development_YAP/TAZ-mediated co-regulation of transcription	5.27E-07	TEF-1, Bcl-XL, Beta-catenin, TEF-5, KLF5, TAZ, Lef-1, YAP1 (YAp65), RUNX2
**13**	Immune response_IFN-gamma signaling via PI3K and NF-kB	5.27E-07	PDK (PDPK1), Beta-catenin, Fyn, PI3K cat class IA, c-Raf-1, GSK3 beta, RelA (p65 NF-kB subunit), NF-kB, SP1

**14**	Growth factors in regulation of oligodendrocyte precursor cells survival in multiple sclerosis	5.91E-07	Bcl-XL, PDK (PDPK1), IRS-1, Fyn, PI3K cat class IA, c-Raf-1, RelA (p65 NF-kB subunit), IBP
**15**	Development_TGF-beta-dependent induction of EMT via RhoA, PI3K and ILK	1.23E-06	RhoA, PDK (PDPK1), Beta-catenin, TGF-beta receptor type II, PI3K cat class IA, GSK3 beta, RelA (p65 NF-kB subunit), Lef-1
**16**	PI3K signaling in gastric cancer	2.38E-06	PDK (PDPK1), HGF receptor (Met), Beta-catenin, IRS-1, CBL-B, PI3K cat class IA, GSK3 beta, RelA (p65 NF-kB subunit)
**17**	HBV-dependent NF-kB and PI3K/AKT pathways leading to HCC	2.38E-06	NF-kB p65/c-Rel, PDK (PDPK1), PI3K cat class IA, c-Raf-1, GSK3 beta, RelA (p65 NF-kB subunit), NF-kB, CDK2
**18**	Development_Growth factors in regulation of oligodendrocyte progenitor cell proliferation	2.51E-06	PDK (PDPK1), HGF receptor (Met), IRS-1, Fyn, PI3K cat class IA, KV1.5, c-Raf-1, GSK3 beta, PKC
**19**	Development_Thrombopoietin signaling via ERK1/2 and PI3K	2.51E-06	PDK1, Bcl-XL, PDK (PDPK1), BMP4, PI3K cat class IA, c-Raf-1, GSK3 beta, RelA (p65 NF-kB subunit), SP1
**20**	Stellate cells activation and liver fibrosis	3.65E-06	MyD88, Beta-catenin, Tcf(Lef), TGF-beta receptor type II, PI3K cat class IA, IRAK1/2, c-Raf-1, GSK3 beta, SP1
**21**	Androgen receptor activation and downstream signaling in Prostate cancer	3.78E-06	K-RAS, Bcl-XL, PDK (PDPK1), PI3K cat class IA (p110-beta), KLF5, IRS-1, c-Raf-1, Kallikrein 2, IBP3, Kallikrein 3 (PSA), VIL2 (ezrin)
**22**	Immune response_B cell antigen receptor (BCR) pathway	3.78E-06	GSK3 alpha/beta, CKLFSF7, K-RAS, c-Rel (NF-kB subunit), Bcl-XL, PDK (PDPK1), c-Raf-1, GSK3 beta, RelA (p65 NF-kB subunit), NF-kB, CARD11
**23**	Cell adhesion_Classical cadherin-mediated cell adhesion	4.88E-06	Vinculin, Beta-catenin, Cortactin, p120-catenin, Alpha-actinin, Plakoglobin
**24**	Main growth factor signaling cascades in multiple myeloma cells	6.61E-06	GSK3 alpha/beta, K-RAS, PDK (PDPK1), IRS-1, PI3K cat class IA, c-Raf-1, NF-kB
**25**	Cytoskeleton remodeling_Regulation of actin cytoskeleton organization by the kinase effectors of Rho GTPases	7.53E-06	RhoA, Vinculin, RhoA-related, Cortactin, ERM proteins, MyHC, Alpha-actinin, Spectrin
**26**	Signal transduction_AKT signaling	9.2E-06	GSK3 alpha/beta, Bcl-XL, PDK (PDPK1), HGF receptor (Met), IRS-1, PI3K cat class IA, NF-kB
**27**	Nicotine / nAChR alpha-7 signaling in NSCLC	9.2E-06	AP-2A, PKC-lambda/iota, PDK (PDPK1), PI3K cat class IA, c-Raf-1, CDK2, SP1
**28**	Apoptosis and survival_nAChR in apoptosis inhibition and cell cycle progression	9.63E-06	GSK3 alpha/beta, PDK (PDPK1), Fyn, PI3K cat class IA, c-Raf-1, GSK3 beta
**29**	Immune response_M-CSF-receptor signaling pathway	1.01E-05	RhoA, PDK (PDPK1), Beta-catenin, Tcf(Lef), Fyn, PI3K cat class IA, c-Raf-1, NF-kB, PKC
**30**	IGF signaling in lung cancer	1.08E-05	Bcl-XL, PDK (PDPK1), IRS-1, PI3K cat class IA, c-Raf-1, IBP, IBP3

Table S7: Univariate and multivariate Cox proportional hazards regression analysis of PDAC overall survival (OS) outcome. Factors showing significant relationship with OS from univariate analysis were then used for multivariate analysis from breast TCGA database. HR, hazard ratio; CI, confidence interval; *: p values < 0.05.

	Overall Survival			
**Univariate**	Patient number (N)	HR	(95% CI)	P value
**Age**				
**<60**	55	ref		
**≧60**	122	1.392	(0.8835-2.192)	0.154
**Gender**				
**Female**	80	ref		
**Male**	97	0.8142	(0.5405-1.227)	0.326
**Tumor_Stage**				
**Stage I - II**	165	ref		
**Stage III -IV**	8	0.7231	(0.2279-2.295)	5.82E-01
**Treatment**				
**No**	86			
**Yes**	70	1.233	(0.7894-1.927)	0.357
**M_Stage**				
**M0**	79	ref		
**M1**	4	0.9275	(0.2237-3.845)	0.917
**MX**	94	0.8247	(0.5423-1.254)	0.368
**T_Stage**				
**T1 - T2**	30	ref		
**T3 - T4**	146	2.156	(1.113-4.176)	0.0228*
**OSBPL3**				
**Low**	88	ref		
**High**	89	1.579	(1.038-2.402)	0.0329*
**OSBPL5**				
**Low**	89	ref		
**High**	88	1.16	(0.7684-1.75)	0.481
**OSBPL6**				
**Low**	89	ref		
**High**	88	0.8517	(0.5655-1.283)	0.442
**OSBPL8**				

**Low**	90	ref		
**High**	87	1.138	(0.7543-1.717)	0.538
**OSBPL10**				
**Low**	89	ref		
**High**	88	1.728	(1.14-2.62)	0.01*
**OSBPL11**				
**Low**	88	ref		
**High**	89	1.064	(0.7058-1.605)	0.766
**Multivariate**				
**T_Stage**				
**T1 - T2**	30	ref		
**T3 - T4**	146	1.884	(0.9540-3.721)	0.0681
**OSBPL3**				
**Low**	88	ref		
**High**	89	1.198	(0.7598-1.889)	0.4366
**OSBPL10**				
**Low**	89	ref		
**High**	88	1.502	(0.9664-2.335)	0.0706

Table S8: Multivariate analysis of OSBPL expression and relationships between it and clinicopathological parameters (age, treatment, stage, and TNM (tumor, node, metastasis) stage)

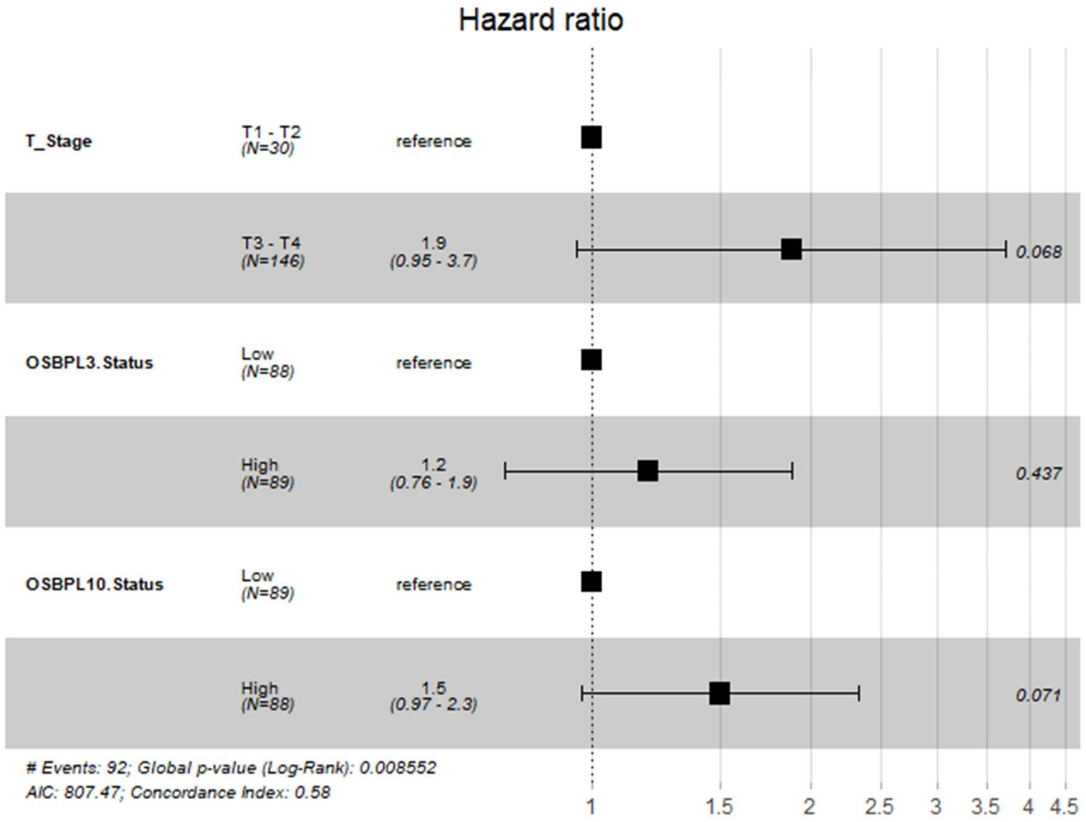

Supplement: Supplementary file 1 [file biomedicines-09-01601-s001.zip › biomedicines-1382810-supplementary.pdf]
